# IL-4 Inhibits IL-1*β*-Induced Depressive-Like Behavior and Central Neurotransmitter Alterations

**DOI:** 10.1155/2015/941413

**Published:** 2015-08-31

**Authors:** Hyun-Jung Park, Hyun-Soo Shim, Kyungeh An, Angela Starkweather, Kyung Soo Kim, Insop Shim

**Affiliations:** ^1^Acupuncture and Meridian Science Research Center (AMSRC), Department of Science in Korean Medicine, Graduate School, College of Korean Medicine, Kyung Hee University, 1 Hoegi-dong, Dongdaemun-gu, Seoul 130-701, Republic of Korea; ^2^Department of Psychology and Center for Neuroscience, Brigham Young University, Provo, UT 84602, USA; ^3^Department of Neuroscience and Cell Biology, University of Texas Medical Branch, Galveston, TX 77551, USA; ^4^Department of Adult Health and Nursing Systems, School of Nursing, Virginia Commonwealth University, Richmond, VA 23298-0567, USA; ^5^Center for Advancement in Managing Pain, School of Nursing, University of Connecticut, Storrs, CT 06269, USA; ^6^Department of Integrative Medicine and Research Center of Behavioral Medicine, College of Medicine, The Catholic University of Korea, Seoul 137-701, Republic of Korea

## Abstract

It has been known that activation of the central innate immune system or exposure to stress can disrupt balance of anti-/proinflammatory cytokines. The aim of the present study was to investigate the role of pro- and anti-inflammatory cytokines in the modulation of depressive-like behaviors, the hormonal and neurotransmitter systems in rats. We investigated whether centrally administered IL-1*β* is associated with activation of CNS inflammatory pathways and behavioral changes and whether treatment with IL-4 could modulate IL-1*β*-induced depressive-like behaviors and central neurotransmitter systems. Infusion of IL-4 significantly decreased IL-1*β*-induced anhedonic responses and increased social exploration and total activity. Treatment with IL-4 markedly blocked IL-1*β*-induced increase in PGE_2_ and CORT levels. Also, IL-4 reduced IL-1*β*-induced 5-HT levels by inhibiting tryptophan hydroxylase (TPH) mRNA and activating serotonin transporter (SERT) in the hippocampus, and levels of NE were increased by activating tyrosine hydroxylase (TH) mRNA expression. These results demonstrate that IL-4 may locally contribute to the regulation of noradrenergic and serotonergic neurotransmission and may inhibit IL-1*β*-induced behavioral and immunological changes. The present results suggest that IL-4 modulates IL-1*β*-induced depressive behavior by inhibiting IL-1*β*-induced central glial activation and neurotransmitter alterations. IL-4 reduced central and systemic mediatory inflammatory activation, as well as reversing the IL-1*β*-induced alterations in neurotransmitter levels. The present findings contribute a biochemical pathway regulated by IL-4 that may have therapeutic utility for treatment of IL-1*β*-induced depressive behavior and neuroinflammation which warrants further study.

## 1. Introduction

Cytokines have been implicated in the evolution of several neuropathological states, including depressive disorders. Like stressors, which may engender features of depressive disorder in rodents, the systemic administration of proinflammatory cytokines such as interleukin-1*β* (IL-1*β*) and tumor-necrosis factor-*α* (TNF-*α*) increases hypothalamic-pituitary-adrenal (HPA) activity [1, 2]. Additionally, cytokines increase monoamine turnover in several limbic sites [3–5]. Another important cytokine is IL-4, which is a kind of anti-inflammatory cytokine. Although it is normally not synthesized at high levels in the brain, it is strongly expressed during brain injury or infection [6]. Several studies reported that anti-inflammatory cytokines have the ability to suppress the synthesis of IL-1, TNF, and other cytokines in peripheral immune and nonimmune cells [7]. However, the different regulatory functions of cytokines and the effect on behavioral changes and monoamine levels in animal models of depression are currently under investigation.

In the present study, we aimed to investigate the role of pro- and anti-inflammatory cytokines in the modulation of depressive-like behaviors and the hormonal and neurotransmitter systems. We sought to determine (1) whether centrally administered IL-1*β* is associated with activation of CNS inflammatory pathways and depressive-like behavioral changes using the tail suspension test, social interaction, and sucrose intake; (2) whether IL-1*β*-induced behavioral changes are associated with peripheral inflammatory pathways, prostaglandin E_2_ (PGE_2_), or corticosterone (CORT); and (3) whether treatment with IL-4 modulates IL-1*β*-induced depressive-like behaviors and central neurotransmitter systems by measuring the concentration of serotonin (5-HT) and norepinephrine (NE) after IL-1*β* intracerebroventricular (i.c.v.) injection.

## 2. Materials and Methods

### 2.1. Animals

All the experimental procedures performed on the animals were conducted with the approval of the Ethics Committee of Kyung Hee University and in accordance with the US National Institutes of Health “Guide for the Care and Use of Laboratory Animals” (NIH Publication number 80-23, revised 1996). Sprague-Dawley rats (Orient Animal Corp., Kyunggi-do, Korea) that weighed 220–240 g each were used for the experiments. The male rats were group-housed (three per cage) under a reversed light-dark cycle (light on from 08:00 to 20:00 hr). The room temperature was 20~25°C and the humidity was 30 ± 5%. The rats had free access to food and water. All the rats were handled daily for at least a week prior to the experiment.

### 2.2. Surgery and Intracranial Drug Injections

Rats were anesthetized with sodium pentobarbital (50 mg/kg, i.p.) and placed in a stereotaxic apparatus. The skull was firmly placed in the apparatus and the scalp was shaved and cleaned with betadine. An incision was made through the skin and muscle to expose the skull and the skin was then retracted. Guide cannulae, 22-gauge, aimed at terminating 1 mm above the 3rd ventricle (AP-0.8 mm, ML-0.5, DV-6 mm), were stereotaxically implanted using dental cement with three screws to secure them to the scull. The cannulae were lowered in the sagittal plane following retraction of the superior sagittal sinus. A 28-gauge stainless steel obturator which extended 1 mm beyond the end of the guide cannula was then inserted. Following surgery, sterile penicillin (1 cc/kg, Durapen) was given to all rats. The rats were allowed 7 days to recover from surgery before testing.

Intracerebroventricular (i.c.v) infusion of rat recombinant IL-1*β* (Sigma) or IL-4 (Sigma) was performed into the ventricle through the guide cannula over a time course of 5 min using a 2 uL/min syringe pump (CMA 102, CMA Microdialysis, Solna, Sweden) connected to PE-10 tubing (Plastic One, Pennsylvania, USA) precut to the appropriate length. The injector (Plastic One) was left in place for another 2 hr to allow for drug diffusion. The injector extended 1.0 mm below the end of the guide cannula into the ventricle. All the employed coordinates were from the atlas of Paxinos et al. [8].

Rats received microinjections of rat recombinant IL-1*β* at the 3rd ventricle (100 ng) or autologous CSF (CSF group, *N* = 5) as healthy control group. Two hours later the animals injected with IL-1*β* were given i.c.v. injections of either 100 ng (*N* = 6) or 200 ng (*N* = 6) of IL-4 or saline (vehicle group, *N* = 6) in the volume of 0.5 uL.

### 2.3. Sucrose Intake and Body Temperature

The animals were transported to a testing room, to which they were allowed to adapt for 1 hr prior to testing. For the sucrose intake test, subjects were trained to consume 1% sucrose solution prior to the start of the experiment. They were exposed to 1% sucrose solution for a 48 h period in their home cage without any food or water available. Testing took place once, between 14:00 and 15:00 hr. Prior to the test, animals were food and water deprived for 20 hr. Sucrose solution consumption was recorded by reweighing preweighed bottles of test solution [9]. Body temperature was measured 7 hours after IL-1*β* i.c.v. injection.

### 2.4. Tail Suspension Test (TST)

A short piece of paper adhesive tape (about 6 cm) was attached along half the length of the tail (about 3 cm). The free end of the adhesive tape was attached to a 30 cm long rigid tape (made from the paper tape folded several times) which was attached to a seesaw lever inked to a spring strain gauge that activated the hand of a spring balance. The animal was surrounded by white-painted wooden enclosed arms (*H*: 54 cm, *W*: 30 cm, and *D*: 47 cm), such that the rat's head was about 10 cm above the floor. Rats were observed for 6 min. As recently pointed out by Mayorga and Lucki [10], one of the confounding factors in the tail suspension test is tail-climbing behavior. The tail-climbing periods tend to be scored as immobility by a mechanical device although they clearly constitute avoidance behavior.

### 2.5. Social Exploration

Behavioral observations were carried out during the dark phase of the cycle, under red light illumination. Rats were introduced into the home cage of the test animal for 5 min [11]. One day before the experiment, baseline social exploration was assessed. Therefore, the total time spent by the experimental rat in social exploration during the 5 min session was recorded by a skilled observer blinded to the experimental conditions. Notably, the total time in contact with the juvenile, but only contact that was directly initiated by the experimental rat and defined as “social exploration” (e.g. anogenital and body sniffing, following, and grooming of the juvenile), was recorded. Thus, leaning against the juvenile or incidental side-by-side touching was not counted.

### 2.6. Corticosterone (CORT) and Prostaglandin E_2_ (PGE_2_) Measurement

After the behavior test, we collected blood samples from the rats. The total concentration of CORT and PGE_2_ was measured by an ELISA kit (DuoSet ELISA Development System, R&D Systems, Inc., Minneapolis, MN, USA). Cardiac blood was collected just prior to sacrificing the rats. The blood was centrifuged for 15 minutes at 1000 ×g within 30 minutes of collection. The samples were immediately assayed or stored at ≤−60°C. All the reagents, working standards, and samples were prepared. The excess microplate strips were removed from the plate frame and returned to the foil pouch containing the desiccant pack and then the pouch was sealed. All of the samples or standards (100 uL) were added to the appropriately labeled wells and 50 uL of conjugated serum was placed into all of the wells except for the nonspecific binding wells and the total count wells. CORT or PGE_2_ (50 uL) was added to all of the wells. All of the wells were incubated for two hours at room temperature on a horizontal orbital microplate shaker (0.12′′ orbit) set at 500 ± 50 rpm. Each well was washed three times with wash buffer. After the last washing, any remaining wash buffer was removed by aspirating or decanting it. 5 uL of CORT or PGE_2_ conjugate and 200 uL of* p*-nitrophenyl phosphate-substrate were added to all of the wells. The well was incubated for 1 hour at room temperature (without shaking). Next, 50 uL of Stop Solution was added to each well. Using a microplate reader, the optical density of each well was immediately determined. The absorbance was read at 450 nm and 550 nm, and the sample values were calculated from a standard curve.

### 2.7. Glial Fibrillary Acidic Protein (GFAP) Measurement

Animals were sacrificed after behavioral test and then brain tissues were collected. The total concentration of GFAP in the hypothalamus was measured by an ELISA kit (ELISA Development System, USCN Life Science, Inc., Huston, TX, USA). The brain protein samples were immediately assayed or stored at ≤−70°C. All the reagents, working standards, and samples were prepared. The protein of brain was extracted by RIPA buffers. The excess microplate strips were removed from the plate frame and returned to the foil pouch containing the desiccant pack and then the pouch was sealed. All of the samples or standards (100 uL) were added to the appropriately labeled wells and incubated for 2 hours at 37°C. Detection reagent A was added to each well followed by incubation for one hour at room temperature on a horizontal orbital microplate shaker (0.12′′ orbit) set at 500 ± 50 rpm. Each well was washed three times with wash buffer. After the last washing, any remaining wash buffer was removed by aspirating or decanting it. Following this, 100 uL of detection reagent B was added to each well and the plate was incubated for 30 minutes at room temperature (without shaking). Each well was washed three times with wash buffer. After the last washing, any remaining wash buffer was removed by aspirating or decanting it. Next, 100 uL of substrate working solution was added to each well and incubated for 5–10 minutes at 37°C. Using a microplate reader, the optical density of each well was immediately determined. The absorbance was read at 450 nm and 550 nm, and the sample values were calculated from a standard curve.

### 2.8. Serotonin (5-HT) and Norepinephrine (NE) Measurement

The total concentration of 5-HT and NE in the brain was measured by an ELISA kit (ELISA Development System, Labor Diagnostika Nord, Inc., Minneapolis, MN, USA). First, all samples were acylated in acylation plate for 24 hr. Acylated samples were transferred into a 5-HT antibody coated 96-well plate, 25 uL of 5-HT antiserum was added to each well, and the plate was incubated for 20 hr. Each well was washed three times with wash buffer. After the last washing, any remaining wash buffer was removed by aspirating or decanting it. Following this, 100 uL of enzyme conjugate was added and incubated for 20 min at RT. Next, 100 uL of Stop Solution was added to each well. Using a microplate reader, the optical density of each well was immediately determined. The absorbance was read at 450 nm, and the sample values were calculated from a standard curve.

### 2.9. Tryptophan Hydroxylase (TPH), Serotonin Transporter (SERT), Tyrosine Hydroxylase (TH), and Norepinephrine Transporter (NET) mRNA Measurement by RT-PCR

Total RNA was isolated using TRIzol reagent (Invitrogen Co., Carlsbad, CA, USA) according to the manufacturer's instructions. Reverse transcription was performed with 2 ng of total RNA using PrimeScript RTase (Takara Bio Inc., Shizuoka, Japan) and random hexamer primers. The subsequent amplification of GAPDH, SERT, NET, TH, and TPH was done by PCR in a total volume of 20 uL containing 0.5 U of rTaq polymerase (Takara Bio Inc.) and 10 pmol of a specific primer set. The primers used were as follows: for GAPDH, forward 5′-TGA TGC TGG TGC TGA GTA AGT CGT-3′, reverse 5′-TTG TCA TTG AGA GCA ATG CCA GCC-3′; for SERT, forward 5′-CCA CCT TCC CAT ACA TTG T-3′, reverse 5′-CTG TCT CCA AGA GTT TCT GC-3′; for NET, forward 5′-GGA GTG GGC CTA TGC TGT GAT-3′, reverse 5′-GTC ATG GAT CCC ACT GCT CT-3′; for TH, forward 5′-GGA GCT GAA GGC TTA TGG TG-3′, reverse 5′-CCAT TGA AGC TCT CGG ACA CA-3′; and for TPH, forward 5′-GCT GAA CAA ACT CTA CCC AAC-3′, reverse 5′-TTC CCG ATA GCC ACA GTA TT-3′. The reaction conditions were as follows: 5 min at 95°C followed by 24–32 cycles of 94°C for 30 s, 52–54 for 40 s, and 72°C for 1 min with a final extension at 72°C for 7 min. The amplified products were separated on a 2% agarose gel and stained with ethidium bromide. The results were analyzed using the image analysis program CoreBio i-MAX TM (CoreBio Co., Seoul, Korea).

### 2.10. Data Analysis

All the results from behavioral, biochemical, and immunological tests are expressed as means ± standard error of the mean (SEM). Statistical analyses were performed using SPSS 15.0 software (SPSS Inc., Chicago, IL). One-way analysis of variance (ANOVA) was performed in order to identify statistically significant changes in the biochemical and behavioral data, followed by the post hoc LSD test. Normality of distribution and equality of variances were confirmed using these tests. *P* values ≤ 0.05 were considered statistically significant.

## 3. Results

### 3.1. IL-4 Attenuated IL-1*β*-Induced Body Temperature and Anhedonia Response

**Figure 1:**
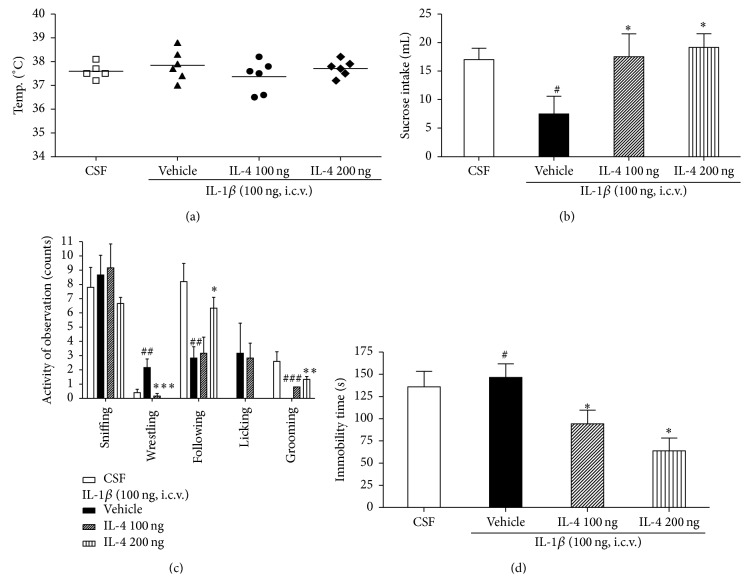
Effect of IL-4 injection 2 hours after injection of i.c.v. IL-1*β* on body temperature (a), sucrose intake (b), social interaction (c), and tail suspension test (d). Treatment effects were assessed on the mean activity (counts) during 5 minutes. The rats were randomly assigned to four groups of six individuals each as follows: CSF group received microinjections of CSF; IL-1*β* group received microinjections of rat recombinant IL-1*β* at the 3rd ventricle; IL-4 groups received microinjections of rat recombinant IL-4 at the 3rd ventricle. Each value represents the mean ± SEM. ^#^ *P* < 0.05, ^##^ *P* < 0.01, and ^###^ *P* < 0.001 compared to the CSF and ^*∗*^ *P* < 0.05, ^*∗∗*^ *P* < 0.01, and ^*∗∗∗*^ *P* < 0.001 compared to the IL-1*β* treated group. Control, *n* = 5; IL-1*β*, *n* = 5; IL-4 100 ng, *n* = 5; IL-4 200 ng, *n* = 5.

Even though IL-1*β* can be a pyrogen itself, at the dose used here and with no provision of additional ambient warmth, we only observed a modest elevation of body temperature of 1°C or less after IL-1*β* injection in rats. After 6 hours, the rat's body temperature returned to the baseline level (Figure 1(a)).

Considering that anhedonia is one of the core symptoms of depression, the effects of IL-1*β* administration with a sucrose intake test were investigated.  Figure 1(b) shows that IL-1*β* (100 ng/site, i.c.v.) administration produced an anhedonic effect as evaluated by the decrease in sucrose intake as compared to the CSF group (*F*_3,22_ = 3.1, *P* < 0.05). Moreover, sucrose intake in IL-4 (i.c.v.) treated rats that were administered IL-1*β* (100 ng/site, i.c.v.) 2 hr later was restored, showing that the anhedonic effects of this cytokine are sensible to anti-inflammatory cytokine treatment. The LSD test results indicate significantly decreased depressive behavior in the IL-4 groups compared to that of the vehicle group (*P* < 0.05).

### 3.2. IL-4 Attenuated IL-1*β*-Induced Reduction in Social Interaction

Figure 1(c) presents the mean number for each group in which rats did active investigation, anogenital sniffing, wrestling, following, licking, and grooming of a juvenile stimulus animal in their home cage during a 5 min test. IL-1*β* administration to the rats led to a robust suppression of social interaction. In particular, active behaviors on the social exploration test were significantly different when compared among the groups (wrestling:* F*_3,22_ = 8.9, *P* < 0.01; following:* F*_3,22_ = 6.5, *P* < 0.01; grooming:* F*_3,22_ = 15.2, *P* < 0.001). The LSD test results indicate markedly decreased social exploration (following and grooming) behavior in the vehicle group as compared to that of the CSF group. However, after IL-4 treatment, rats showed significant increase in the sum of social behaviors (following and grooming) and increase in wrestling number compared to the vehicle group.

### 3.3. IL-4 Attenuated IL-1*β*-Induced Depressive Behavior

We evaluated the ability of rats to cope with a stressful and inescapable situation (learned helplessness) with a tail suspension test (TST). As shown in Figure 1(d), the animals displaying increased immobilization periods were considered to have increased helplessness, which is a sign of depressive-like behavior (*F*_3,22_ = 5.4, *P* < 0.05). When tested with the TST, the vehicle treated rats showed more increased immobility time during the 5 min test than the CSF group. However, after IL-4 treatment, rats displayed significantly decreased immobility time in the TST (*P* < 0.05).

### 3.4. IL-4 Altered IL-1*β*-Induced Increases in Plasma CORT

**Figure 2:**
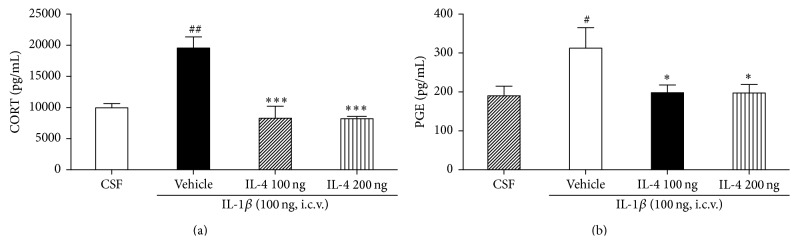
(a) Effect of IL-4 injected 2 hours after injection of i.c.v. IL-1*β* on CORT concentration. Each value represents the mean ± SEM. ^##^ *P* < 0.01 compared to the CSF and ^*∗∗∗*^ *P* < 0.001 compared to the IL-1*β* treated group. Control, *n* = 5; IL-1*β*, *n* = 5; IL-4 100 ng, *n* = 5; IL-4 200 ng, *n* = 5. (b) Effect of IL-4 injection 2 hours after injection of i.c.v. IL-1*β* on PGE_2_ concentration. Each value represents the mean ± SEM. The results of ELISA were analyzed by performing separate one-way ANOVA among the groups. Each value represents the mean ± SEM. ^#^ *P* < 0.05 compared to the CSF and ^*∗*^ *P* < 0.05 compared to the IL-1*β* treated group. Control, *n* = 5; IL-1*β*, *n* = 5; IL-4 100 ng, *n* = 5; IL-4 200 ng, *n* = 5.

Plasma levels of CORT in the IL-1*β* treated group were significantly elevated 6 hr after i.c.v. injection compared with the CSF group (*F*_3,22_ = 15.0, *P* < 0.001; Figure 2(a)). The LSD test results indicated significantly increased serum levels of CORT in the vehicle group compared to the CSF group (*P* < 0.01). However, the treatment of IL-4 resulted in markedly decreased serum levels of CORT compared to the vehicle group (*P* < 0.001).

### 3.5. IL-4 Altered IL-1*β*-Induced Increases in Plasma PGE_2_

Plasma levels of PGE_2_ in the vehicle group were significantly elevated 6 hr after i.c.v. injection compared with the CSF group (*F*_3,22_ = 3.2, *P* < 0.05; Figure 2(b)). The LSD test results indicated significantly increased serum levels of PGE_2_ in the vehicle group compared to the CSF group (*P* < 0.05). However, the treatment of IL-4 led to markedly decreased serum levels of PGE_2_ compared to the vehicle group (*P* < 0.05).

### 3.6. IL-4 Altered IL-1*β*-Induced 5-HT Level and SERT and TPH Expression in the Brain Regions

**Figure 3:**
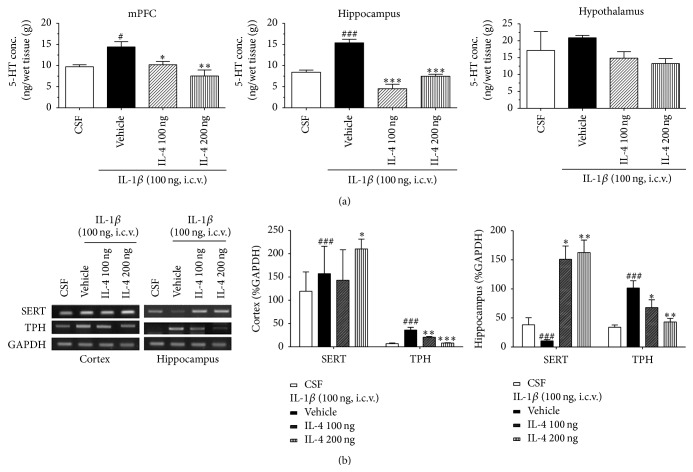
(a) Effect of IL-4 injection 2 hours after injection of i.c.v. IL-1*β* on 5-HT concentration. Mean (±SEM) concentrations (nanogram per gram) of 5-HT within the mPFC, hippocampus, and hypothalamus among rats. Each value represents the mean ± SEM. ^#^ *P* < 0.05, ^###^ *P* < 0.001 compared to the CSF and ^*∗*^ *P* < 0.05, ^*∗∗*^ *P* < 0.01, and ^*∗∗∗*^ *P* < 0.001 compared to the IL-1*β* treated group. Control, *n* = 5; IL-1*β*, *n* = 5; IL-4 100 ng, *n* = 5; IL-4 200 ng, *n* = 5. (b) Effect of IL-4 injection 2 hours after injection of i.c.v. IL-1*β* on SERT and TPH mRNA levels in the brain. Values are means ± SEM. Relative % GAPDH of SERT and TPH mRNA levels within the mPFC and hippocampus among rats. Each value represents the mean ± SEM. ^###^ *P* < 0.001 compared to the CSF, ^*∗*^ *P* < 0.05, ^*∗∗*^ *P* < 0.01, and ^*∗∗∗*^ *P* < 0.001 compared to the IL-1*β* treated group. Control, *n* = 5; IL-1*β*, *n* = 5; IL-4 100 ng, *n* = 5; IL-4 200 ng, *n* = 5.

Central 5-HT level in brain regions was changed after i.c.v. IL-1*β* infusion that has been linked to major depression (Figure 3(a)). In particular, 5-HT levels in the prefrontal cortex and hippocampus were increased by the IL-1*β* treatment (*F*_3,22_ = 6.0 and 13.1, *P* < 0.01). However, both IL-4 treated groups showed appreciably decreased 5-HT levels in the hippocampus and cortex. Within the hypothalamus, 5-HT levels were not affected by the cytokine treatment.

Level of SERT in the vehicle group was significantly increased in the cortex after IL-1*β* injection compared with the CSF group (*F*_3,22_ = 8.2, *P* < 0.01), whereas levels of SERT in the hippocampus were reduced (*F*_3,22_ = 8.4, *P* < 0.01). Treatment with IL-4 markedly potentiated SERT levels in the prefrontal cortex and hippocampus compared with the vehicle group (*F*_3,22_ = 8.2, *P* < 0.01).

In several brain regions (the prefrontal cortex and hippocampus), expression of tryptophan hydroxylase was increased by IL-1*β* injections (*F*_3,22_ = 6.9, *P* < 0.01). Both IL-4 treated groups markedly decreased TPH levels in the prefrontal cortex compared with the vehicle group (Figure 3(b)).

### 3.7. IL-4 Increased i.c.v. IL-1*β*-Induced NE Level and TH and NET Expression in the Brain Regions

**Figure 4:**
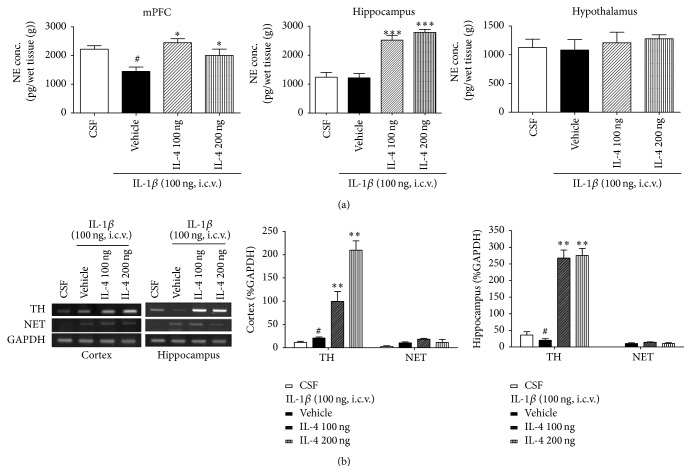
(a) Effect of IL-4 injection 2 hours after injection of i.c.v. IL-1*β* on NE concentration. Mean (±SEM) concentrations (picogram per gram) of NE within the mPFC, hippocampus, and hypothalamus among rats. Each value represents the mean ± SEM. ^#^ *P* < 0.05 compared to the CSF and ^*∗*^ *P* < 0.05, ^*∗∗∗*^ *P* < 0.001 compared to the IL-1*β* treated group. Control, *n* = 5; IL-1*β*, *n* = 5; IL-4 100 ng, *n* = 5; IL-4 200 ng, *n* = 5. (b) Effect of IL-4 injection 2 hours after injection of i.c.v. IL-1*β* on NET and TH mRNA levels in the brain. Values are means ± SEM. Relative % GAPDH of NET and TH mRNA levels within the mPFC and hippocampus among rats. Each value represents the mean ± SEM. ^#^ *P* < 0.05 compared to the CSF and ^*∗∗*^ *P* < 0.01, compared to the IL-1*β* treated group. Control, *n* = 5; IL-1*β*, *n* = 5; IL-4 100 ng, *n* = 5; IL-4 200 ng, *n* = 5.

Central NE level in brain regions was changed after i.c.v. IL-1*β* infusion that has been linked to major depression (Figure 4(a)). In several brain regions (the prefrontal cortex, hippocampus, and hypothalamus), expression of NE was altered by IL-4 treatment (*F*_3,22_ = 6.9 and 32.3, *P* < 0.01, *P* < 0.01, resp.). Both IL-4 treated groups markedly increased the NE level in the prefrontal cortex and hippocampus compared to the vehicle group. However, NE levels in the hypothalamus were not affected by cytokine treatment.

Expression of TH in the vehicle group was significantly increased in the cortex after IL-1*β* injection compared with the CSF group (*F*_3,22_ = 6.9, *P* < 0.01), whereas expression of TH in the hippocampus was reduced (*F*_3,22_ = 6.5, *P* < 0.01). Treatment with IL-4 markedly potentiated TH expressions in the prefrontal cortex and hippocampus compared with the vehicle group (*F*_3,22_ = 7.2, *P* < 0.01).

However, the NET level in the limbic regions was not affected by IL-4 treatment (Figure 4(b)).

### 3.8. IL-4 Altered IL-1*β*-Induced Increases in GFAP

**Figure 5:**
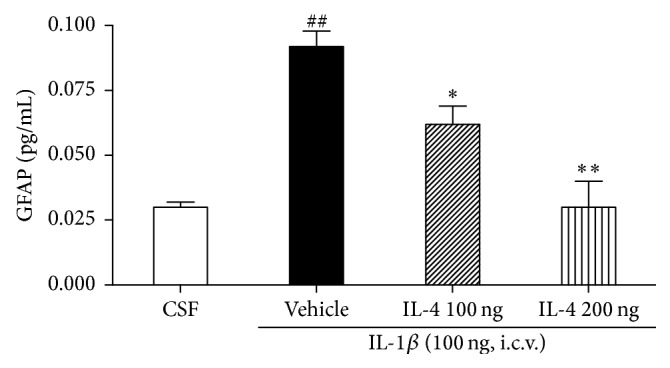
Effect of IL-4 injection 2 hours after injection of i.c.v. IL-1*β* on GFAP concentration. Mean (±SEM) concentrations (nanogram per gram) of GFAP within the hypothalamus among rats. Each value represents the mean ± SEM. ^##^ *P* < 0.01 compared to the CSF, ^*∗*^ *P* < 0.05, and ^*∗∗*^ *P* < 0.01, compared to the IL-1*β* treated group. Control, *n* = 5; IL-1*β*, *n* = 5; IL-4 100 ng, *n* = 5; IL-4 200 ng, *n* = 5.

Levels of GFAP in the vehicle group were significantly elevated 6 hr after i.c.v. injection compared with the CSF group (*F*_3,22_ = 4.2, *P* < 0.05; Figure 5). The LSD test results indicated significantly increased levels of GFAP in the vehicle group compared to the CSF group (*P* < 0.01). However, the treatment of IL-4 led to markedly decreased serum levels of GFAP compared to the vehicle group. We also examined the levels of GFAP in the cortex and hippocampus but did not show changes among groups.

## 4. Discussion

The goal of this experiment was to examine the behavioral and biochemical changes induced by anti-inflammatory cytokine IL-4 after IL-1*β* administration. We have shown that centrally administered IL-4 reduced depressive-like behaviors that were induced by i.c.v. IL-1*β*. Additionally, central administered IL-4 normalized the metabolism of multiple hormones and neurotransmitters, including CORT, the inflammatory mediator PGE_2_, and 5-HT and NE.

Lending clear support to the hypothesis that centrally injected IL-1*β* can cause depressive behaviors, rats administered IL-1*β* i.c.v. demonstrated significant changes in depressive-like behavior. Bluthé et al. [12] observed that the increases of IL-1*β* in the serum were strongly related with the induction of a depressive-like behavior in rats. Systemic lipopolysaccharide (LPS) administration also increased the expression of IL-1*β*. In this study, administration of IL-1*β* produced a decrease in total activity, social exploration, and anhedonic behavior. In a number of previous experiments, 100 ng doses of IL-1*β* have been shown to reduce sweetened milk consumption [13].

The anti-inflammatory action of IL-4 is well documented both* in vitro* and* in vivo*. IL-4 attenuates the activation of various immunocompetent cells, including neutrophils, monocytes, and macrophages, by limiting the production of proinflammatory cytokines [14–16], and it decreases production of PGE_2_ [17]. Exposure of murine peritoneal macrophages to murine IL-4 for 16 hr before stimulation with IL-1*β* induced production of PGE_2_. In addition, IL-1*β* is a potent modulator of corticotrophin-releasing hormone which produces heightened hypothalamic-pituitary-adrenal axis activity characterized by increases in adrenocorticotropic hormone (ACTH) and CORT, both of which are reported to be generally elevated in major depression. A proinflammatory effect of IL-1 observed* in vitro* is induction of PGE_2_ synthesis in fibroblasts [18]. Consistent with previous studies, injection of IL-1*β* significantly increased level of CORT and PGE_2_ in the serum. These results showed that IL-1*β* i.c.v. quickly destroyed peripheral immunological homeostasis. However, several cytokines, such as IL-1*α*, transforming growth factor-*β*, IL-4, and IL-10, have been described as regulating COX-2 induction and PGE_2_ production [19, 20]. Also, observed* in vitro* was suppression of IL-1*β*-induced PGE_2_ generation in mouse osteoblastic cells by IL-4, a Th_2_ cell-derived cytokine. Our data reinforce this inhibitory effect of IL-4 on IL-1*β*-induced PGE_2_ expression by demonstrating the capacity of this cytokine to downregulate IL-1*β*-induced delayed PGE_2_ and CORT biosynthesis in rats.

Glial fibrillary acidic protein (GFAP) is the intermediate filament protein most commonly used as a marker for the identification of astrocytes* in vivo* and* in vitro* and is known to be elevated following neuroinflammatory-induced glial activation. It is characterized by immunogenic determinations [21]. In this study we examined the change of GFAP levels in the hypothalamus. Astrocytes serve as one of the representatives of the immune system in the brain, protecting the brain from invading microorganisms. They are primarily responsible for the inflammatory reaction in response to brain damage. In this study, it is possible that IL-1*β*-induced increases in GFAP may be compensatory mechanisms to repair a decrease of neuroinflammation responses after IL-4 injection [22]. One limitation of the present studies is that only a change of GFAP was examined, since many of these factors would be expected to show the regulatory effect of neuroinflammation-behavior-GFAP activation. The next step will be to evaluate the activation of microglia and anatomically specify these changes at the protein level.

In conclusion, IL-4 has antidepressive effects by reducing immobility time on TST and increasing SERT, NE, and TH mRNA levels in the brain regions. The study provides evidence that IL-4 plays an important role in modulating the hormonal and neurotransmitter regulatory systems that are relevant for IL-1*β*-induced depressive behaviors. The cross talk between these systems, mediated by IL-4, may become a target for novel antidepressant therapies. Moreover, the acute administration of IL-1*β* can be a reliable inflammatory preclinical model of depressive-like behavior that is sensitive to antidepressant treatment that may be useful to test potential new antidepressant drugs. In the future, studies of molecules that might represent a link of the modulation of neural, endocrine, and immune systems are extremely important to further understand the etiology of depression.

## Abbreviations

5-HT: Serotonin
NE: Norepinephrine
IL-4: Interleukin-4
IL-1*β*: Interleukin-1-beta
TPH: Tryptophan hydroxylase
TH: Tyrosine hydroxylase.
